# Tephrosin induces apoptosis of human pancreatic cancer cells through the generation of reactive oxygen species

**DOI:** 10.7150/jca.50360

**Published:** 2021-01-01

**Authors:** Jie Du, Fan Jiang, Shen-Sheng Xu, Zi-Feng Huang, Li‐Li Chen, Li Li

**Affiliations:** 1 Department of Hepatobiliary Surgery, the Affiliated Puren Hospital of Wuhan University of Science and Technology, Wuhan, China.; 2 Department of Centre for clinical teaching skills, the Affiliated Puren Hospital of Wuhan University of Science and Technology, Wuhan, China.

**Keywords:** Tephrosin, pancreatic cancer, reactive oxygen species, apoptosis

## Abstract

Tephrosin is a natural rotenoid isoflavonoid that has been shown to have potent anticancer activities. In this study, we reported the anticancer activity of tephrosin against pancreatic cancer cells. Tephrosin potently suppressed cell viability in various cancer cell lines and promoted apoptosis of PANC-1 and SW1990 pancreatic cancer cells evidenced by enhanced cleavage of caspase-3/-9 and PARP. Further studies showed that tephrosin increased the production of intracellular reactive oxygen species (ROS) and led to mitochondrial membrane potential depolarization, and subsequent cytochrome c release. DNA damage was also identified by increased tail DNA and phosphorylation of H2AX. Intracellular ROS production seems to be essential for the anticancer activity of tephrosin, alleviation of ROS production by ROS scavengers weakened the apoptotic effects of tephrosin. Importantly, in PANC-1 xenografted nude mice, potent antitumor activity and low toxicity of tephrosin were observed. In conclusion, these results indicated that tephrosin could be developed as a potential chemotherapeutic agent for the treatment of human pancreatic cancer.

## Introduction

Pancreatic cancer, one of the most lethal malignancies, is the fourth leading cause of cancer-related death worldwide now, with an estimated 5-year survival rate of <10% 1. It is characterized with early metastasis, delayed diagnosis, weak response to standard chemotherapy, and poor prognosis after treatment. Many patients diagnosed with pancreatic cancer are often asymptomatic at the early stage and receive treatment in an advanced stage 2. Thus, few patients can undergo surgical resection, and chemotherapeutic treatment which is a common choice for advanced patients 3. Gemcitabine has been the first line chemotherapy drug for the treatment of pancreatic cancer for more than 10 years 4. However, only modest survival (7-8 months) could benefit from gemcitabine in pancreatic cancer patients, novel safer drugs with improved efficacy are still in urgent need 5.

ROS have significant oxidative activity and affect many cellular biological processes, including proliferation, invasion, apoptosis and cell death 6. The doubled-edged sword role of ROS in tumor cells has been recognized gradually in recent years 7. At low concentrations, ROS act as intracellular messengers and facilitate cell survival and progression, while large amount of ROS production causes apoptosis and leads to damage of various cellular organelles, finally leading to the cell death 8. Generated ROS influence mitochondrial membrane permeability, which triggers a series of mitochondrial associated events 9. In addition, excessive ROS can also result in oxidative DNA damage 10. Particularly, increased studies revealed the pivotal roles of ROS in pancreatic cancer, making it a potential target to develop drugs for pancreatic cancer therapy 11.

**Figure 1:**
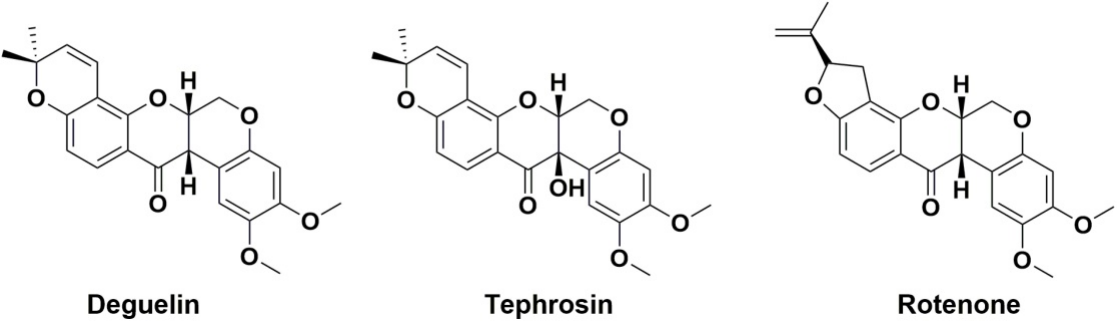
Chemical structures of deguelin, tephrosin and rotenone.

Chinese herbs have long been used for the treatment of various cancers and many natural products have been reported to inhibit pancreatic cancer cell growth by inducing apoptosis and blocking the generation of ROS 12, 13. Deguelin, tephrosin and rotenone (**Fig. 1**) are rotenoid isoflavonoids isolated from plant genus *Derris* 14. Rotenoids are known to have anticancer and insecticidal activities, and especially, prominent antitumor activity of deguelin and its anticancer mechanisms have been widely reported, but less in the anticancer study of tephrosin 15, 16. Tephrosin has been reported to suppress mouse skin tumor growth and induce internalization of EGFR and ErbB2 in colon cancer cells 17. Although rotenoids are attractive compounds to develop antitumor chemotherapeutics 18, their therapeutic effects on pancreatic cancer and the detailed mechanisms associated with the anticancer activity of tephrosin are still largely unknown.

In the present study, we explored the antitumor activity and mechanisms of tephrosin against pancreatic cancer cells. Our data showed that tephrosin exhibited potent antitumor activity against pancreatic cancer cells and increased intracellular ROS levels and subsequent apoptosis. Our study also demonstrated the promising antitumor activity of tephrosin *in vivo*.

## Materials and methods

### Drugs and reagents

Deguelin, tephrosin and rotenone were purchased from MedChemExpress (Monmouth Junction, NJ, USA). These compounds were dissolved in dimethyl sulfoxide directly at a concentration of 20 mM and stored at -20 °C for use. Cell culture reagents were obtained from Life Technologies (Grand Island, NY, USA). DCF-DA, MTT, JC-1 and other reagents, unless otherwise indicated, were all purchased from Sigma-Aldrich (Millipore, Darmstadt, Germany).

### Cell lines and cell cultures

Human cancer cell lines A549, MCF-7, HepG2, SHG-44, SW1990, PANC-1, CFPAC-1 and MIAPaCa were purchased from the cell bank of Shanghai Biological Institute, Chinese Academy of Sciences (Shanghai, China). A549 cells were cultured in F12K medium, HepG2 cells were cultured in minimum essential medium (MEM), and other cell lines were cultured in DMEM. Medium for all cell lines contains 10% fetal bovine serum (FBS), 1% penicillin-streptomycin (Gibco, Grand Island, NY, USA) and all cells were maintained in a humidified atmosphere of 5% CO_2_ at 37 °C. HUVEC were maintained in ham's F-12, supplemented with 10% FBS, heparin and endothelial cell growth factor. Cells at the mid-log phase were used in the experiments.

### Cell viability assay

5×10^3^ Cells were seeded into every well of 96-microwell plates, following attachment, cells were treated with various concentrations of tephrosin. After treatment for indicated time points, MTT solution was added and incubated at 37 °C for 4 h. The medium was removed and 100 μL DMSO was added and absorbance was quantified at 490 nm with a microplate reader (Bio-Tek, Vermont, USA). For blocking study, cells were pretreated with 5 mM NAC, 25 μM Z-VAD-FMK for 1 h.

### Colony formation assay

About 500 PANC-1 or SW1990 cells were seeded into 6-well plates and then treated with different concentrations of tephrosin for 24 h. After treatment, cells were washed with fresh medium and permitted to form cell colonies for another 7 days. The cell colonies were washed once with PBS and fixed with methanol for 15 min, and then stained with 0.1% crystal violet for 15 min. After washing with PBS for 3 times, the stained colonies were photographed using Huawei P30 smartphone (Huawei, China).

### Hoechst 33258 staining

1×10^5^ Cells were seeded into 6-well plats. After attachment, cells were treated with various concentrations of tephrosin for 24 h. After treatment, the cells were washed with PBS for three times and incubated with 20 μg/mL Hoechst 33258 at room temperature for 15 min in the dark. After incubation, stained cells were observed under a fluorescent microscope (Olympus, Tokyo, Japan).

### Annexin V/FITC-PI assay

Annexin V-FITC/PI apoptosis detection kit was used to perform the apoptotic assay following the manufacturer's instructions (Beyotime, Jiangsu, China). Briefly, after treatment, 1×10^6^ cells were washed using 1 mL binding buffer for three times, then centrifuged at 300×g and stained with 10 μL Annexin V-FITC solution at 37 °C for 15 min. Before detecting, 5 μL PI solution was added to the samples and the apoptotic cells were detected using flow cytometry (BD Biosciences, USA).

### Caspase-3 activity measurement

The activity of caspase-3 was detected using the Caspase-3 Activity Assay Kit (Beyotime, Jiangsu, China). The absorbance (A405) was measured using an ELISA reader after treatment with tephrosin for 12 h (Bio-Tek, Vermont, USA).

### Mitochondrial membrane potential assay

1×10^5^ Cells were seeded into 6-well plats. After attachment for 12 h, cells were treated with various concentrations of tephrosin for 24 h. Then, cells were incubated with 10 µg/mL JC-1 dye for 30 min at 37 °C in the dark and then washed with PBS buffer for 5 min. After incubation, samples were immediately detected for red and green fluorescence by a microplate reader (Bio-Tek, Vermont, USA) or fluorescence microscopy (Olympus, Tokyo, Japan).

### Detection of intracellular ROS

1×10^5^ Cells were seeded into 6-well plats. ROS levels were measured using the ROS Assay Kit (Beyotime, Jiangsu, China). In brief, cells were treated with tephrosin for different time points, then incubated with 10 μM 2,7-dichlorodihydrofluorecein diacetate (DCF-DA) for 20 min at 37 °C. PBS was used to wash the cells for 3 times before detection. Fluorescence microscope (Olympus, Tokyo, Japan) was used for direct observation. To detect fluorescence intensity, cells were collected and fluorescence was detected with a flow cytometry (BD Biosciences, USA).

### Western blot analysis

1×10^5^ Cells were seeded into 6-well plats. Cells after treatment were lysed and centrifuged at 12,000×g for 15 min. Proteins were separated using SDS-polyacrylamide gel and electrotransferred to PVDF membranes. The membranes were initially blocked with 5% nonfat dry milk and then probed with primary antibodies against pro-caspase-3, cleaved caspase-3, pro-caspase-9, cleaved caspase-9, cleaved PARP-1, cytochrome c, p-H2A.X or GAPDH, which were diluted following the manufacturer's instructions at 4 °C overnight. Then the membranes were blotted with the horseradish peroxidase-conjugated anti-mouse or anti-rabbit secondary antibodies for 2 h at 37 °C. Finally, the bands were visualized through the enhanced chemiluminescence protocol.

### *In vivo* study

Six-week-old BALB/c nude mice were purchased from Shanghai Experimental Animal Center of Chinese Academy of Science (Shanghai, China). All animal protocols were implemented according to the guidelines of the association for Assessment and Accreditation of Laboratory Animal Care. 3×10^6^ PANC-1 cells were subcutaneously implanted into the right flank of mice. After 6 d of tumor growth, tumors grew to approximately 100 mm^3^ and mice were randomly divided into three groups with six animals in each group. Mice in two groups were intraperitoneally injected tephrosin (10 and 20 mg/kg, respectively) every day for a total of 13 days. The control group was intraperitoneally injected an equal volume of DMSO. Body weights and tumor volumes were measured every two days. After treatment, all of the mice were sacrificed and the tumors were harvested, weighted and photographed. The tumor volume was calculated according the formula: Tumor volume = (length×width^2^)/2.

### Statistical Analyses

All data are given as mean ± SD and analyzed by one-way ANOVA. Statistical significance was determined as *P* < 0.05. All data were obtained from three independent experiments.

## Results

### Tephrosin effectively induced cytotoxicity of pancreatic cancer cells

To compare the cytotoxicity of deguelin, tephrosin, and rotenone, their antiproliferative activities were evaluated on various cancer cell lines, including A549, MCF-7, HepG2, SHG-44 and SW1990 by an MTT assay. Fig. 1A-C indicated that pancreatic cancer cells SW1990 were more sensitive to tephrosin. The half-maximal inhibitory concentrations (IC_50_) of tephrosin against several pancreatic cancer lines were further investigated and the detailed values as follows: 2.62 μM (SW1990), 0.82 μM (PANC-1), 2.91 μM (CFPAC-1), and 2.79 μM (MIAPaCa) (**Fig. 1D-E**). Interestingly, both human normal pancreatic cells (HPC-Y5) and human umbilical vein endothelial cells (HUVEC) were not sensitive to tephrosin and the IC_50_ values are 41.21 μM and 18.86 μM, respectively. Furthermore, tephrosin induced cell death in a dose- and time-dependent manner (**Fig. 1F-G**), and colony formation was also inhibited in a concentration-dependent manner in both PANC-1 and SW1990 cells (**Fig. 1H**).

### Tephrosin induced apoptosis of pancreatic cancer cells

**Figure 3:**
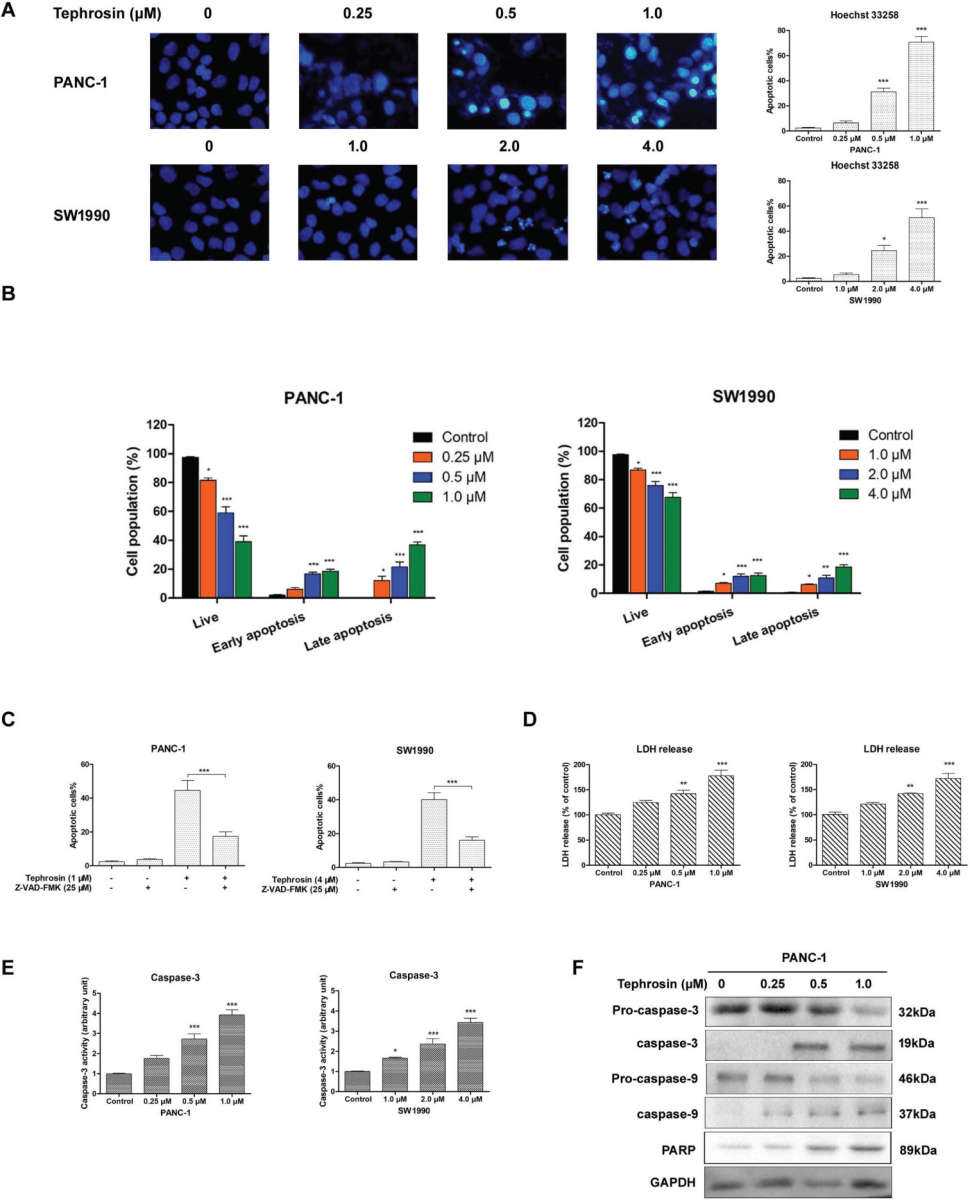
** Tephrosin induced apoptosis of pancreatic cancer cells.** (**A**) Cell apoptosis was detected by Hoechst 33258 staining. Cells with condensed or fragmented nuclei after treatment of tephrosin were identified as apoptotic cells. (**B**) Apoptosis of PANC-1 and SW1990 cells were detected by Annexin-V/PI co-staining followed with flow cytometric analysis. (**C**) Effects of the caspase inhibitor Z-VAD-FMK (25 µM) preatment on tephrosin-induced apoptosis in PANC-1 and SW1990 cells. (**D**) LDH was detected to evaluate the apoptotic response to treatment of tephrosin in PANC-1 and SW1990 cells. (**E**) Caspase-3 activity increased after treatment with tephrosin at indicated concentrations for 12 h. (**F**) Treatment of tephrosin up-regulated the expresssion of clevaed caspase-3, caspase-9, and PARP. Data represented as the mean ± SD (standard deviation) of three independent experiments. *p < 0.05, **p < 0.01, ***p < 0.001 vs. control group.

The morphology alterations of pancreatic cancer cells after incubation with different concentrations of tephrosin for 24 h were detected by using Hoechst 33258, the chromatin condensation and fragmentation indicating apoptosis in nuclei were observed in PANC-1 and SW1990 cells (**Fig. 3A**). Annexin-V/PI co-staining were further performed to confirm the apoptosis induced by tephrosin. Treatment of PANC-1 cells with 0.5 μM and 1.0 μM of tephrosin induced cell apoptosis to 31.2% and 68.3%, respectively (**Fig. 3B**). Pre-incubation with pan-caspase suppressor Z-VAK-FMK significantly ameliorated tephrosin-triggered apoptosis in PANC-1 and SW1990 cells (**Fig. 3C**). The leakage of lactate dehydrogenase (LDH) into the medium is an indication of impairment of cell membrane integrity. Caspase-3 is a critical executioner of apoptosis 19. The increased LDH leakage and caspase-3 activity also identify the apoptotic effects of tephrosin in pancreatic cancer cells (**Fig. 3D-E**). Proteolytic processing of caspae-3, caspase-9 and PARP were further analyzed by Western blot assay to investigate the mechanism by which tephrosin induced apoptosis. Results in **Fig. 3F** showed that treatment of PANC-1 cells with tephrosin induced proteolysis of caspae-3, caspase-9 and PARP. These results implied that tephrosin promoted cell apoptosis of pancreatic cancer cells.

### Tephrosin elevated intracellular ROS levels in pancreatic cancer cells

**Figure 4:**
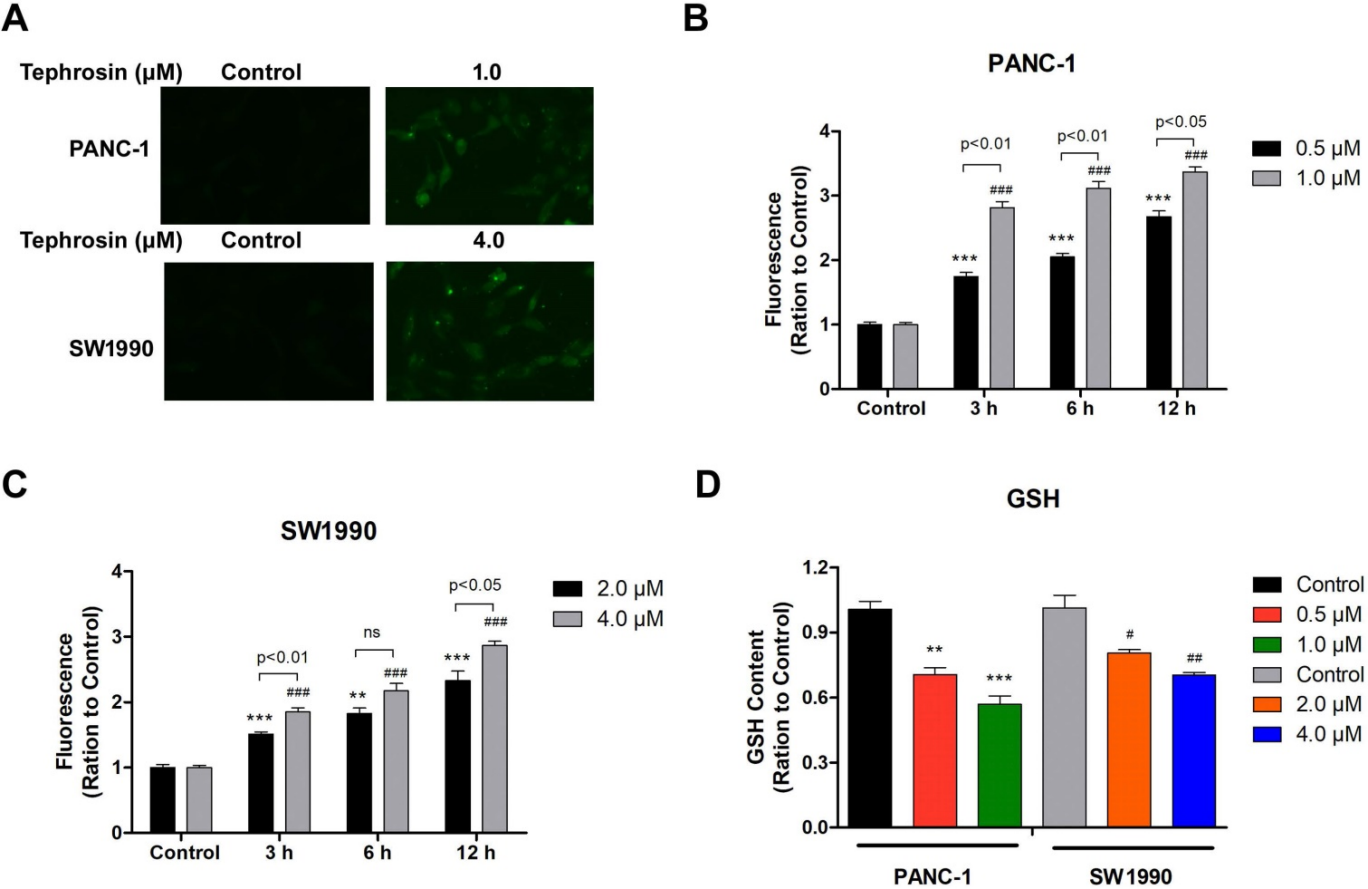
** Tephrosin caused ROS generation in human pancreatic cancer cells.** (**A**) The levels of ROS in PANC-1 and SW1990 cells were measured by DCF-DA staining 1 h after treatment of tephrosin (1 or 4 µM). The green fluorescence intensity is associated with the degree of ROS. (**B, C**) Intracellular ROS accumulated in a dose- and time-dependent manner in tephrosin-treated PANC-1 and SW1990 cells. (**D**) Tephrosin caused a depletion of intracellular levels of GSH. Data represented as the mean ± SD (standard deviation) of three independent experiments. *p < 0.05, **p < 0.01, ***p < 0.001 vs. control group.

Excessive free radical production is known to induce cell apoptosis, thus, fluorescence dye DCF-DA was used to detect whether ROS was induced by tephrosin under fluorescence microscopy. The results showed that tephrosin treatment in PANC-1 and SW1990 cells quickly led to the generation of ROS within 1 hour, indicated by bright green fluorescence (**Fig. 4A**). Further detailed study showed that tephrosin treatment increased ROS accumulation in cells in a time- and dose-dependent manner in both PANC-1 and SW1990 cell lines (**Fig. 4B-C**). The levels of endogenous antioxidants, such as glutathione (GSH), were also detected, and the result showed that tephrosin made a remarkable depletion of intracellular GSH levels in a dose-dependent manner (**Fig. 4D**). These findings suggest that tephrosin induced ROS accumulation in pancreatic cancer cells.

### Tephrosin-induced apoptosis relied on ROS generation

**Figure 5:**
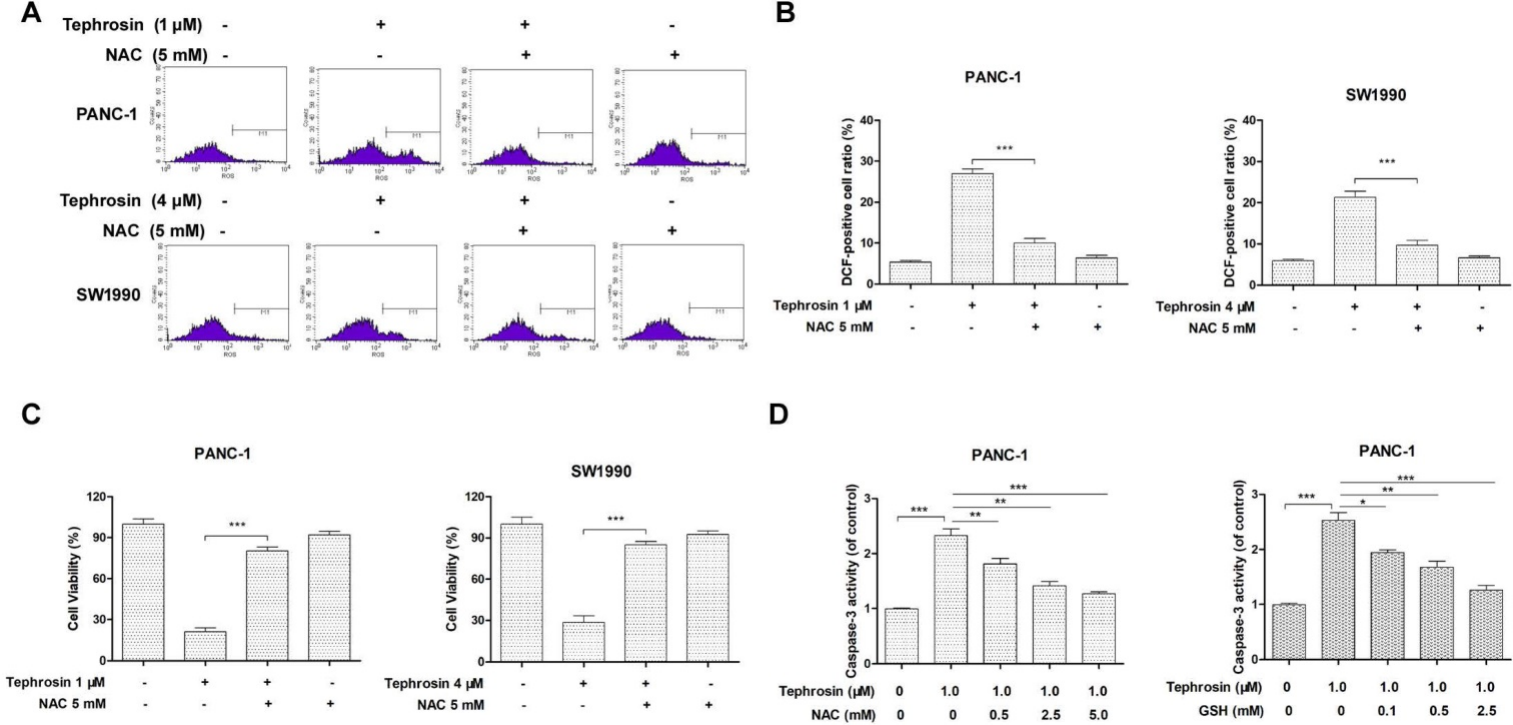
** Tephrosin induced apoptosis in PANC-1 and SW1990 cells was dependent on ROS, pretreatment with antioxidants NAC or GSH for 1 h prior to tephrosin exposure prevented tephrosin-induced ROS accumulation, proliferation, and caspase-3 activation.** (**A**) ROS generation was detected using flow cytometry after staining with DCF-DA. (**B**) Results are expressed as a percentage of relative fluorescence of DCF-DA to the control. The cell viability (**C**) and activity of caspase-3 (**D**) were detected with or without pre-incubation of NAC or GSH. Data represented as the mean ± SD (standard deviation) of three independent experiments. *p < 0.05, **p < 0.01, ***p < 0.001.

In order to clarify the role of ROS in tephrosin-induced apoptosis in pancreatic cancer cells, the cells were pre-treated with ROS scavenger *N*-acetyl-L-cysteine (NAC) for 1 hour, before incubation with tephrosin. DCF staining and flow cytometry were applied to detect the ROS formation. The results in **Fig. 5A-B** showed that the elevated ROS generation in tephrosin-treatment group was abolished by the pre-treatment of NAC in both PANC-1 and SW1990 cells. In addition, NAC pre-incubation eliminated tephrosin-induced apoptosis, evidenced by increased cell viability (**Fig. 5C**) and reduced caspase-3 activity (**Fig. 5D**). Altogether, above results suggested that the apoptosis induced by tephrosin in pancreatic cancer cells might be dependent on ROS generation.

### Tephrosin induced apoptosis through the mitochondrial pathway

**Figure 6:**
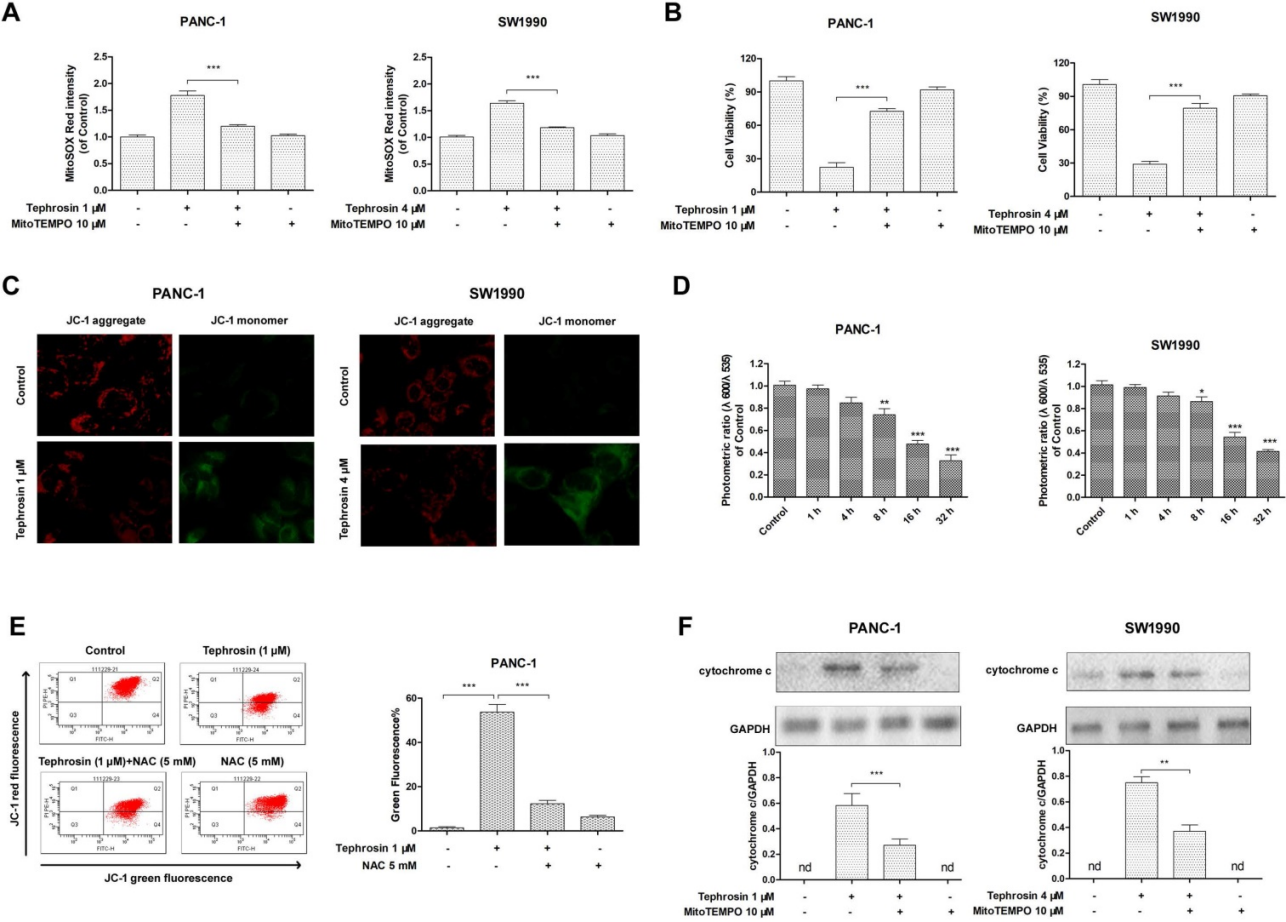
** Effects of tephrosin on mitochondria depolarization in PANC-1 and SW1990 cells.** (**A**) Cells were pre-incubated with MitoTEMPO (10 µM) for 1 h, followed by incubation with tephrosin. The fluorescence intensity of MitoSOX Red was assessed using flow cytometry. (**B**) The cell viability was detected with MTT method. (**C**) Fluorescence photomicrograph of changes in the MMPs indicated by staining with JC-1. (**D**) Decreased photometric ratio of red to green fluorescence indicated the depolarization of MMP. (**E**) Cells with different treatment were stained with JC-1, followed by flow cytometric analysis. (**F**) The release of cytochrome c from the mitochondria was detected by using Western blot. Data represented as the mean ± SD (standard deviation) of three independent experiments, ***p < 0.001.

Mitochondrial membrane depolarization is related to the mitochondrial production of ROS. To confirm whether tephrosin-induced ROS was associated with mitochondria, a specific mitochondrial-ROS scavenger, MitoTEMPO, was used for pre-incubation. The ROS levels in mitochondria were specifically assessed by the indicator, MitoSOX Red. The results showed that tephrosin treatment elevated MitoSOX Red intensity, and MitoTEMPO pre-incubation blunted this elevation (**Fig. 6A**). Tephrosin-induced cytotoxicity was also affected by MitoTEMPO (**Fig. 6B**). The mitochondrial membrane potentials (MMPs) of PANC-1 and SW1990 cells with or without tephrosin were then examined by using JC-1 as a mitochondrial fluorescent probe. In healthy cells, JC-1 aggregates in the mitochondria and exhibited red fluoresce, while in unhealthy cells, JC-1 forms monomer and stains the cytosol green. As shown in **Fig. 6C**, treatment of tephrosin significantly induced the dissipation of MMP in PANC-1 and SW1990 cells. And the ratio of fluorescent intensity of red to green, which indicates cell healthy status, decreased in a time-dependent manner (**Fig. 6D**). Furthermore, the number of unhealthy PANC-1 cells with collapsed MMPs was determined by using a flow cytometric analysis, the result showed that incubation with 1 μM tephrosin yielded 52.5% cells with collapsed MMPs, and pre-incubation of NAC significantly abolished its effects (**Fig. 6E**). Additionally, the release of cytochrome c from the mitochondria to cytosol was observed in PANC-1 and SW1990 cells after incubation with tephrosin (**Fig. 6F**). Taken together, tephrosin has a significant effect on MMPs.

### Tephrosin induced DNA damage through the generation of ROS

**Figure 7:**
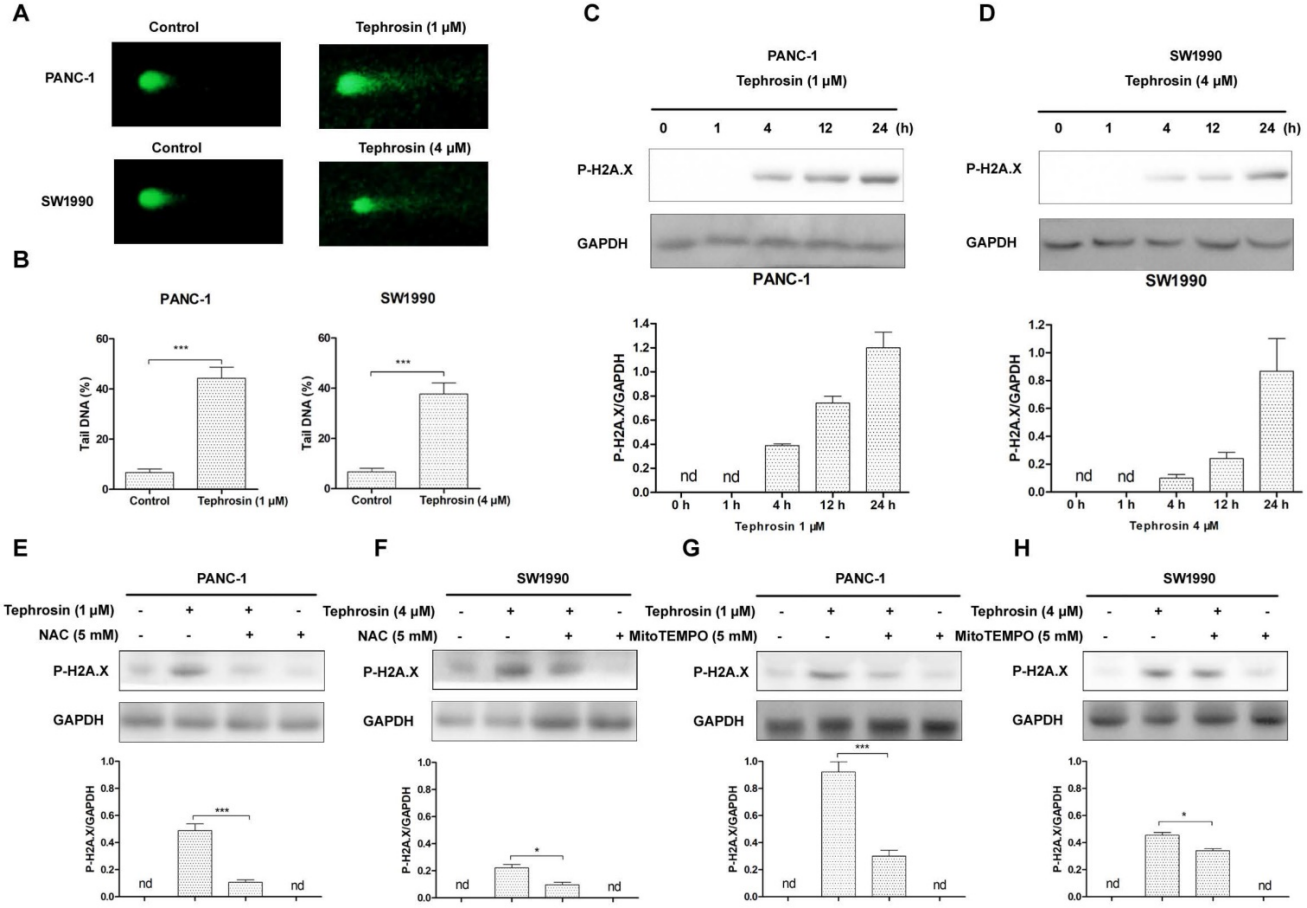
** Tephrosin-induced ROS caused DNA damage in PANC-1 and SW1990 cells.** (**A**) Tephrosin induced DNA stand breaks. PANC-1 and SW1990 cells were treated with tephrosin for 24 h, followed by alkaline comet assay. (**B**) Comet assay results are graphed as means ± SD of percent tail DNA from 20 cells at 3 independent gels. (**C**) Cells were treated with tephrosin for different time points, and the expression of p-H2A.X was detected using Western blot. (**E-H**) Cells were pre-incubated with NAC or MitoTEMPO for 1 h, followed by tephrosin treatment for 24 h. The expression of of p-H2A.X was detected using Western blot. Data represented as the mean ± SD (standard deviation) of three independent experiments. *p < 0.05, ***p < 0.001.

DNA is a primary target of ROS, in order to further investigate whether tephrosin could trigger ROS-mediated oxidative DNA damage, we assessed DNA strand breaks by using the alkaline comet assay 20. As shown in **Fig. 7A**, tephrosin was able to cause DNA stand breaks after 24 h treatment in PANC-1 and SW1990 cells. The DNA stand breaks were quantified by percent of tail DNA (**Fig. 7B**). H2A.X immunoblotting was further performed due to the fact that phosphorylated H2A.X is a sensitive marker of DNA damage. The results in **Fig. 7C-D** showed that H2A.X phosphorylation was increased over time in tephrosin-treated cells. Pre-incubation of ROS scavenger NAC and MitoTEMPO significantly reduced the accumulation of H2A.X phosphorylation in both PANC-1 and SW1990 cells, suggesting the essential role of ROS in tephrosin-induced DAN damage (**Fig. 7E-H**).

### Tephrosin reduced *in vivo* pancreatic cancer growth

**Figure 8:**
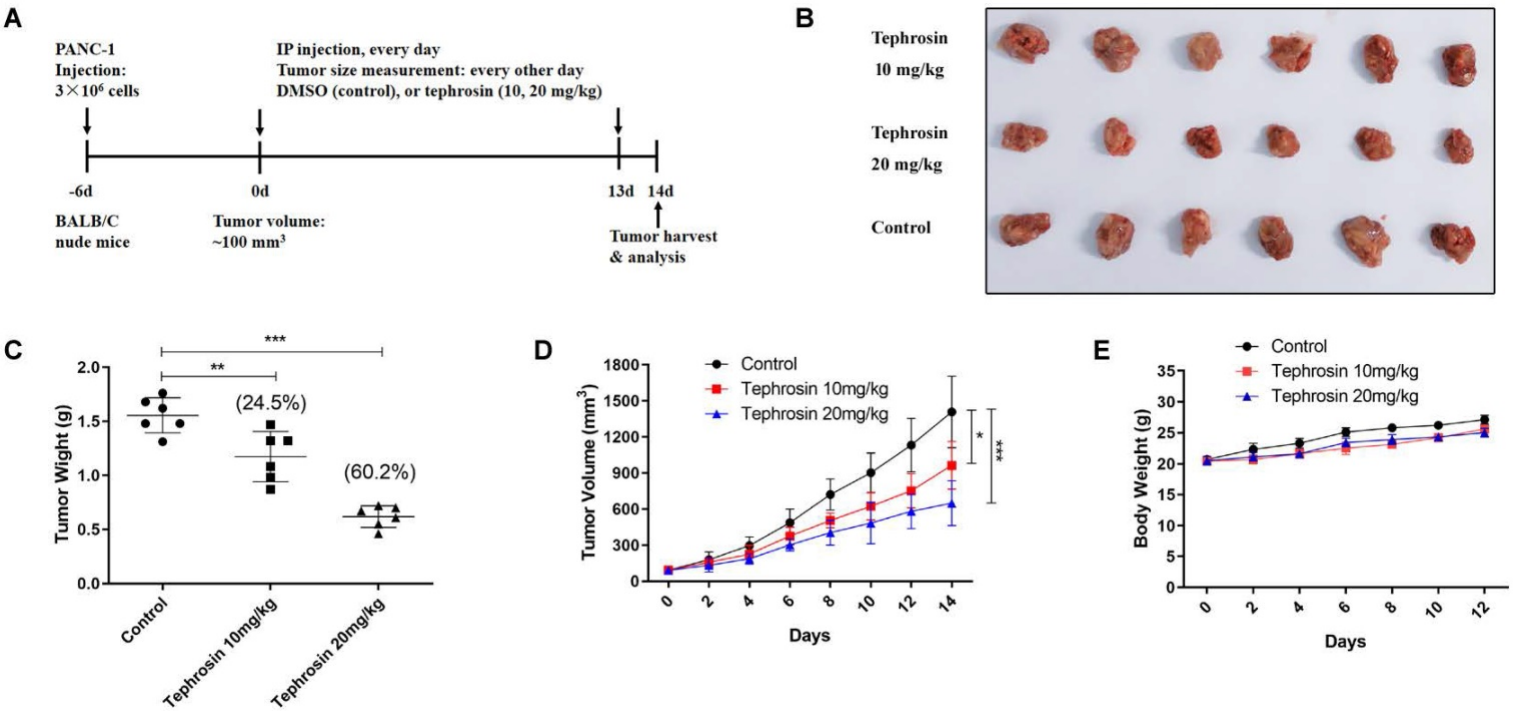
** Tephrosin suppressed tumor growth in PANC-1 xenografted nude mice.** (**A**) Schematic diagram displaying the time course of treatment in mice. (**B**) Representative tumor images from each group after treatment. (**C**) Treatment of tephrosin resulted in significant lower tumor weight compared with control (n =6). (**D**) Tephrosin treatment significantly inhibited the pancreatic cancer cell growth in nude mice. (**E**) No obvious body weight changes of mice between the control and tephrosin-treated groups was observed (n =6). *p < 0.05, **p < 0.01, ***p < 0.001 vs. control group.

**Figure 9:**
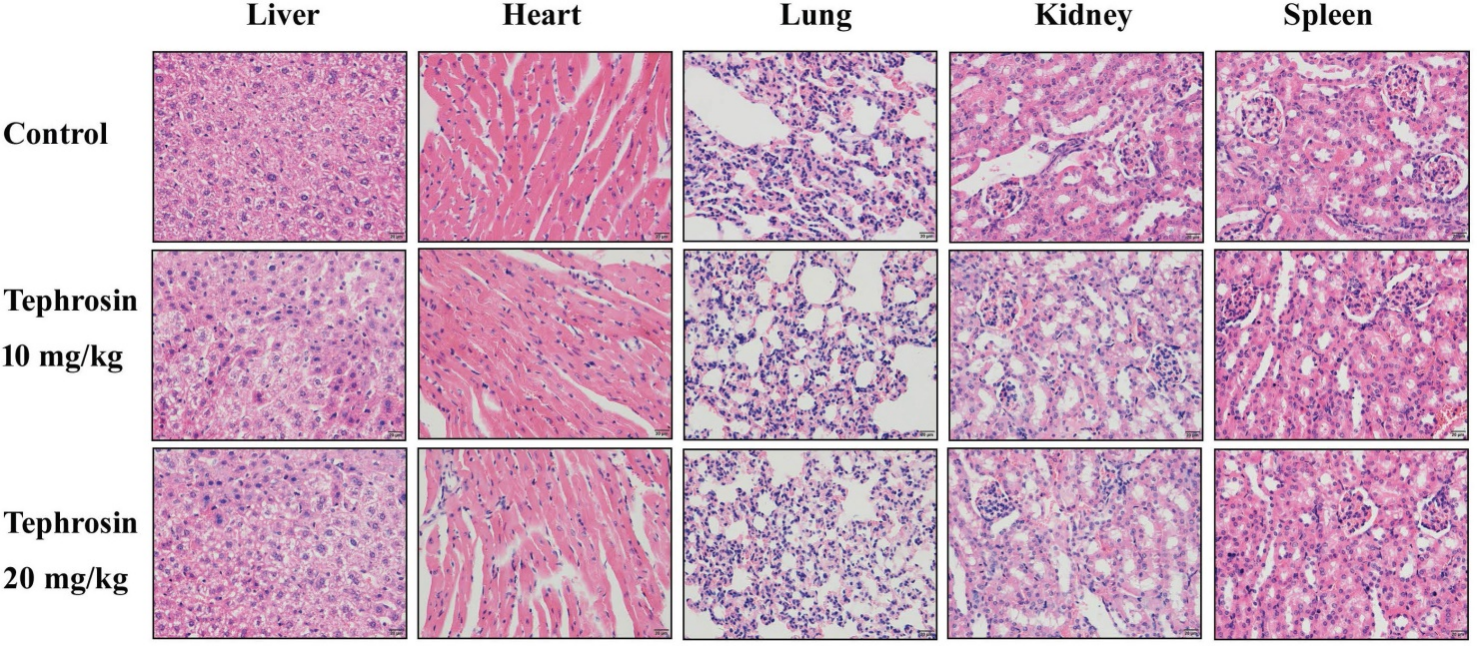
** H&E staining of the important tissues in each group.** The mice were treated with tephrosin or control for two weeks, after sacrificing the mice, the organs were taken out for pathological analysis.

In order to detect the *in vivo* antitumor effects of tephrosin, PANC-1 cells were subcutaneously injected into BALB/C nude mice. After a week of cancer cell transplantion, mice were weighted and divided into three groups, followed by intraperitoneal injection of tephrosin (10 mg/kg and 20 mg/kg) every day for 2 weeks. The tumor size and body weight of the mice were monitored and recorded every 2 days (**Fig. 8A**). By the end of the study, the mice were sacrificed and the excised tumors were shown in Fig. 8B. The statistic results indicated that high-dose tephrosin (20 mg/kg) significantly reduced tumor growth *in vivo* and reached a 60.2% reduction compared with administration of vehicle (**Fig. 8C**). Measurement of the tumor volume showed time-dependent increase of tumors during the observation period in vehicle-treated group, but tephrosin significantly reduced the growth of tumors (**Fig. 8D**). During the treatment period, no obvious body weight changes were observed, suggesting low toxicity of tephrosin (**Fig. 8E**). In addition, H&E staining of important organs, like liver, heart, kidney, lung, and spleen also suggested that no observable pathological changes could be observed in these organs (**Fig. 9**). These data identified the potent *in vivo* antitumor effect and low toxicity of tephrosin against pancreatic cancer.

## Discussion

As one of the most fatal malignancies, pancreatic cancer represents the fourth cause of cancer mortality globally. However, the detailed mechanisms and pathogenesis of pancreatic cancer are still unclear now, which largely hampers the development of tumor targeted drugs. The lack of early diagnosis methods makes it difficult to perform the surgical resection in pancreatic cancer patients and chemotherapy (mainly gemcitabine) is one of the compromises in clinical treatment now, although severe drug resistance has limited its therapeutic efficacy 21. Thus, novel effective drugs are required to improve the clinical outs of pancreatic cancer patients. In recent years, some studies revealed that ROS plays an important role in pancreatic cancer and we supposed that this may be a breakthrough point in developing novel therapeutic drugs. ROS are often accumulated in a little higher level in pancreatic cancer cells than in normal cells, which serves as a signaling intermediate to enhance cellular activity and progression 13. However, excessive release of ROS in cells disrupts the integrity of mitochondria and promotes the release of cytochrome c into cytoplasm and triggers apoptosis of cells. Thus, the balance of ROS levels in pancreatic cancer cells determines the fate of cells.

Considering the important dual role of ROS in cancer cells, increasing the ROS level would be an effective way for cancer treatment. In fact, many compounds, especially natural products, have been reported to cause pancreatic cancer cell death by targeting ROS. For example, some natural compounds, such as sophoridine 22, moscatilin 23, nimbolide 12, and bufalin 24, have been reported to be able to induce pancreatic cancer cell apoptosis by increasing intracellular ROS levels. In addition, many natural products have been identified to exert their antitumor activity in other kinds of cancer cells by targeting ROS 25. Thus, exploring nature-derived compounds might be an effective way to find therapeutic agents against pancreatic cancer. In the present study, three rotenoids, including deguelin, tephrosin and rotenone were explored, and the results showed these compounds have good cytotoxicity against pancreatic cancer cells. Cubé resins have long been used as pesticides and insecticides since the 1850s. Rotenone, deguelin and tephrosin are the most prominent and active ingredients of Cubé resins 26. Although rotenone exhibited more potent antitumor activities than tephrosin and degulin in our study, which was in consistent with previous reports, high toxicity of rotenone ascribed to its inhibition of NADH/ubiquinone oxidoreductase machinery limited its further application 27. The cell viability assay in this study revealed that tephrosin markedly inhibited the growth of various cancer cell lines, and was more sensitive to pancreatic cancer cells with the lowest IC_50_ value of 0.82 μM against PANC-1 cells, implying that tephrosin might be a promising anticancer agent for the treatment of pancreatic cancer. Furthermore, tephrosin has low toxicity to human normal cells, indicating a favorable therapeutic index.

Our study demonstrated that apoptosis is responsible for the anticancer activity of tephrosin. The pre-incubation of a pan-caspase inhibitor, Z-VAK-FMK, significantly eliminated the tephrosin-induced cell death. Furthermore, the expression levels of cleaved caspase-3, caspase-9, and cleaved PARP indicated that tephrosin induced cell death through apoptosis. Substantial studies have identified that ROS participate in the process of apoptosis and several signaling cascades are involved in the ROS-induced apoptosis. Cheng et al reported that Longikaurin E induced apoptosis of pancreatic cancer cells via activating caspase-3 and decreasing the ratio of Bcl-2/Bax mediated by increased ROS 28. Spiclomazine was reported to induce the apoptosis of pancreatic cancer cells through activating caspase-3/caspase-9 cascade mediated through increased ROS 29. Sustained activation of phosphorylation of EKK/JNK was reported to be involved in the sophoridine-induced apoptosis through generation of ROS 22. Zhang et al also reported that moscatilin induced apoptosis of pancreatic cancer cells via JNK/SAPK pathway, which was dependent on ROS generation 23. These studies imply that apoptosis induced by elevated ROS levels is the main anticancer mechanism of many chemotherapies. In this study, we demonstrated that treatment with tephrosin resulted in an increase in ROS generation and pre-treatment of NAC could abolish tephrosin-inhibited cell viability, implying that ROS is responsible for the apoptotic effects of tephrosin on pancreatic cancer cells.

## Conclusion

In this study, the *in vitro* and *in vivo* antitumor activities of tephrosin in pancreatic cancer were evaluated, and its molecular mechanism was also investigated. Tephrosin significantly inhibited the proliferation of pancreatic cancer cells and induced mitochondrial-related apoptosis. ROS are required for tephrosin to exhibit antiproliferative activity and trigger apoptosis in pancreatic cancer cells. Tephrosin significantly inhibited the growth of pancreatic cancer cells *in vivo* and has no observable toxicity, indicating that tephrosin is a potential anticancer agent, and deserves further development as a new therapy for pancreatic cancer.

## Figures and Tables

**Figure 2:**
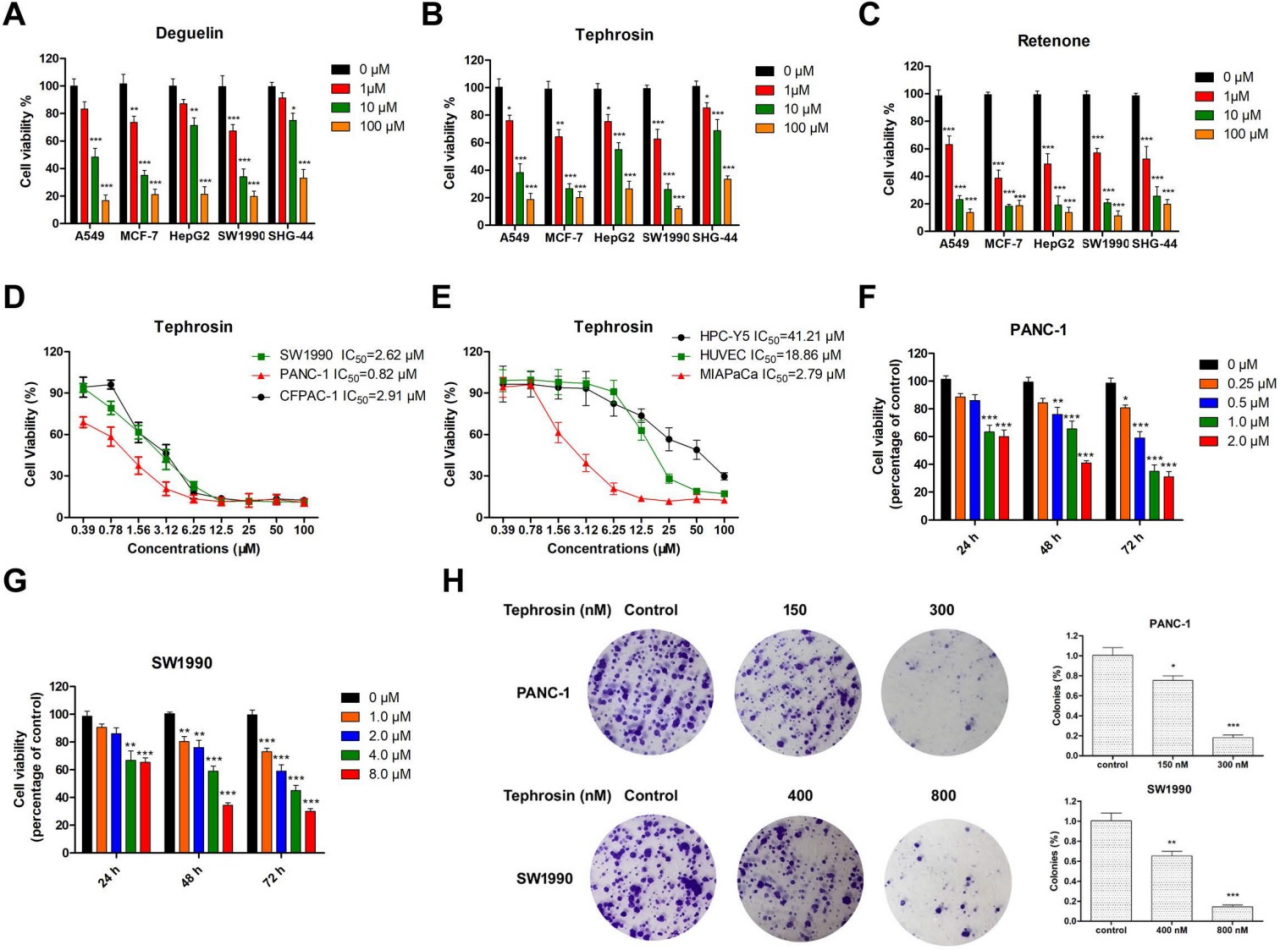
** Tephrosin inhibited the proliferation of various cancer cell lines, and induced pancreatic cancer cell death.** (**A, B, and C**) Results from MTT assay showed that the cellular viabilities of human tumor cells A549 (lung), MCF-7 (breast), HepG2 (liver), SW1990 (pancreas), and SHG-44 (glioblastoma) cells were decreased after the treatment with deguelin, tephrosin, and rotenone at indicated concentrations for 72 h. (**D, E**) MTT assay was used to determine IC_50_ values of tephrosin against pancreatic cancer and normal cell lines. (**F, G**) Tephrosin inhibited the growth of PANC-1 and SW1990 cells in a dose- and time-dependent manner. (**H**) Representative images of the colony formation in PANC-1 and SW1990 cells treated with indicated concentrations of tephrosin for a week, and statistical analysis of the colony numbers. Data represented as the mean ± SD (standard deviation) of three independent experiments. *p < 0.05, **p < 0.01, ***p < 0.001 vs. control group.
